# SQUAMOUS CELL CARCINOMA IN THE FOOT: CASE SERIES AND LITERATURE REVIEW

**DOI:** 10.1590/1413-785220182602187183

**Published:** 2018

**Authors:** IROCY GUEDES KNACKFUSS, VINCENZO GIORDANO, ALEXANDRE LEME GODOY-SANTOS, Nurimar Conceição Fernandes, OLAVO PIRES DE CAMARGO

**Affiliations:** 1. Department of Orthopedics and Traumatology, Faculdade de Medicina da Universidade Federal do Rio de Janeiro, Rio de Janeiro, RJ, Brasil (In memoriam).; 2. Prof. Nova Monteiro Orthopedics and Traumatology Service, Hospital Municipal Miguel Couto, Rio de Janeiro, RJ, Brazil.; 3. Department of Orthopedics and Traumatology, Hospital das Clinicas HCFMUSP, Faculdade de Medicina, Universidade de São Paulo, SP, Brazil.

**Keywords:** Carcinoma, squamous cell, Foot, Amputation, Metastasis., Carcinoma de células escamosas, Pé, Amputação, Metástase.

## Abstract

**Objectives:**

To report a case series of squamous cell carcinoma (SCC) in the foot, describing previous risk factors, surgical treatment, histopathological findings, and functional and oncological results.

**Methods:**

Nine consecutive patients diagnosed with SCC of the foot were treated at a single institution and prospectively analyzed for risk factors related to the disease, surgical outcome, and histopathological, functional and oncological results. All patients had identifiable risk factors for SCC.

**Results:**

Definitive treatment consisted of partial (6 patients) or total (3 patients) amputation of the foot. The functional score was good or excellent in the surviving patients. Early identifiable risk factors are present in most patients. Biopsy when this diagnosis is suspected, in association with oncological principles, avoids diagnostic and treatment errors.

**Conclusion:**

Despite delayed diagnosis and surgical treatment with partial and total amputations of the foot in our series, we observed good oncological outcomes that avoided systemic spread of the disease and achieved expected functional results. Level of Evidence V; Case series.

## RESUMO

**Objetivo:**

. Relatar uma série de casos de carcinoma epidermoide no pé, descrever os fatores de risco prévios, os achados histopatológicos, o tratamento cirúrgico e os resultados funcionais e oncológicos.

**Métodos:**

Nove pacientes consecutivos com diagnóstico de CE na região do pé foram tratados em uma única instituição e analisados, prospectivamente com relação aos fatores de risco da doença, ao desfecho do tratamento cirúrgico e aos resultados histopatológicos, funcionais e oncológicos. Todos os pacientes apresentavam fatores de risco identificáveis.

**Resultados:**

O tratamento definitivo consistiu em amputação parcial (seis), amputação total (três). O escore funcional foi bom ou excelente nos pacientes sobreviventes. Fatores de risco identificáveis precocemente estão presentes na maioria dos pacientes. A biópsia na suspeita diagnóstica e o uso de princípios oncológicos evitam erros de diagnóstico e de tratamento.

**Conclusão:**

Em nossa série, apesar do diagnóstico tardio e do tratamento cirúrgico com amputações parciais e totais do pé, observamos bons resultados oncológicos que evitaram a disseminação sistêmica da doença e resultados funcionais esperados. Nível de Evidência V; Série de casos.

## INTRODUCTION

Squamous cell carcinoma (SCC) is a rare condition in the foot.^1^ ^,^ ^2^ The disease was first described in 1828 by Marjolin, and its malignancy was recognized by Dupuytren.^3^ ^,^ ^4^ SCC originates in keratinocytes and may develop a precursor lesion or de novo lesions.^4^ Verrucous carcinoma, which is not strongly malignant but still locally invasive and destructive, rarely leads to metastases.^2^ ^,^ ^4^ When untreated, the lesions may grow to large diameters. In the plantar region, they are irregularly shaped, well-demarcated, verrucous, and are also known as epithelioma cuniculatum.^5^ Reports in the literature state that 13% occur in the legs, and this is the most common primary cancer of soft tissue in the foot, with an incidence slightly greater than melanoma and synovial sarcoma.^6^

Despite numerous publications on the subject in the literature, many orthopedic physicians demonstrate a lack of familiarity with this condition in their case reports, which invariably delays diagnosis and optimal treatment.^2^ ^,^ ^5^ ^-^ ^13^ Treatment is usually palliative and includes aggressive and broad resection of the tumor.^8^ ^-^ ^10^ Metastatic disease and recurrence of the lesion are uncommon, and are largely associated with incomplete initial excision of the tumor.^11^

The objectives of this study are to report a series of cases of SCC in the foot and to describe previous risk factors, histopathological findings, surgical treatment, and functional and oncological outcomes.

## MATERIALS AND METHODS

The study included patients who were surgically treated for primary SCC of the foot over a 2-year period. The study excluded all patients with metastases related to the SCC at the time of admission, patients with SCC of the skin in other areas of the body, and patients with any other type of benign or malignant skin disease in the ankle/foot region.

The patients sought orthopedic treatment via referral from the Department of Dermatology (n=8) or spontaneous request (n=1). The main complaint was the presence of chronic lesions on the foot, which did not heal, occasionally bleeding and not permitting the patients to use closed shoes. Two patients had secondary infections, with purulent drainage at the time of the first medical visit.

**Figure 1:**
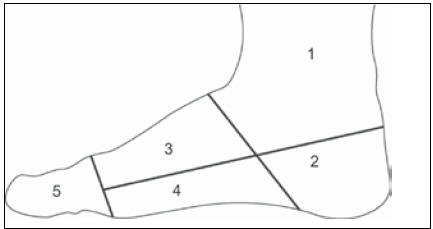
Zones, according to Kirby et al.

Data were collected on patient age, sex, predisposing factors from external conditions, duration of symptoms, location and size of the lesion, tumor staging, and definitive surgical treatment.^14^ ^,^ ^15^ Postoperative complications were analyzed, along with functional results according to the Musculoskeletal Tumor Society Score (MSTS)^16^ and oncological outcomes (remission, local recurrence, metastasis, and death from the disease). The MSTS score is described in Table 1. The location of the lesion was described according to the areas defined by Kirby et al.^17^ (Figure 1)

**Table 1:** Description of MSTS score.

Score	Pain	Function	Emotional	External support	Functional Independence	Gait	Percentage (%)
5	None	No restrictions	Motivated	None	Independent	Normal	100
4	Intermediate	Intermediate	Intermediate	Intermediate	Intermediate	Intermediate	80
3	Not disabling	Recreational limitation	Satisfied	Orthesis	Limited	Minimal alteration	60
2	Intermediate	Intermediate	Intermediate	Intermediate	Intermediate	Intermediate	40
1	Incapacitating	Partial limitation	Accepts	Cane/crutch	Home	Very altered	20
0	Extremely incapacitating	Total restriction	No support	Two crutches	Dependent	Difficult	0

All patients were subjected to routine laboratory examinations and X-rays. A computed tomography scan of the abdomen was performed in two patients with metastasis to the regional lymph nodes. Incisional biopsy was performed routinely, and after the diagnosis was confirmed, the patients were surgically treated as follows: resection of the metatarsal ray was performed in 4 patients, the hallux was amputated in 2 patients, transmetatarsal amputation was performed in 1 patients, and transtibial amputation in 2 patients.

We informed the patients that data relating to these cases would be published, and they provided consent (study was approved by institutional review board under number 6070/3004).

### Statistical analysis

The results were analyzed using descriptive statistics for all variables. The potential link between the duration of symptoms and lesion size was assessed using Pearson correlation analysis, with p<0.05. Potential differences between recurrence rates (recurrence or metastasis) and functional score based on primary definitive treatment were evaluated by Fisher’s exact test, with p<0.05. Statistical analysis was performed using SPSS v.15.0 software (SPSS Inc., Chicago, IL).

## RESULTS

Nine patients were included in the study, eight men and one woman. Mean patient age at treatment was 60 years (range: 45-86 years). Seven individuals were Caucasians and two were Black. In seven cases the tumor was located in the forefoot (Kirby zone 5), one case on the dorsum of the midfoot (Kirby zone 3), and one case on the midfoot plantar region (Kirby zone 4). The fourth toe was affected most often. The time interval between the first symptoms and the first visit ranged from six to 120 months, with an average of 30.7 months. The clear imbalance in this variable resulted from an extremely late diagnosis in case 9, with 120 months of evolution. The average lesion size in its largest linear dimension was 5.2 cm (range: 3.0-8.0 cm). There was no statistical correlation between the time until diagnosis and lesion size (p>5%). Among the predisposing factors, all patients had some social or professional activity related to sun exposure and six (66.7%) smoked more than one pack of cigarettes per day. The demographic data for the patients included in the study are shown in Table 2.

**Table 2:** Demographic data for the study population.

Case	Age	Sex	Race	Evolution time (months)	Location	Predisposing factor(s)
1	72	M	C	12	Right hallux	SE, tobacco use
2	44	F	B	24	Left 2nd toe	SE
3	78	M	C	07	Left 4th toe	SE
4	57	M	C	24	Right hallux	SE, tobacco use
5	40	M	C	06	Left foot, dorsum.	SE, tobacco use
6	86	M	C	24	Left 4th and 5th toes	SE, tobacco use
7	56	M	B	24	Right hallux	SE, tobacco use
8	49	M	C	36	Left 4th toe	SE
9	55	M	C	120	Bottom of right foot	SE, tobacco use

Source: DOT-UFRJ, 2017. Abbreviations: M - male, F - female, C - Caucasian, B - Black, SE - sun exposure.

**Figure 2:**
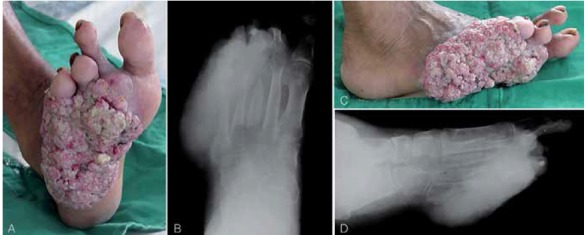
Case 9. Squamous cell carcinoma, ulcerative type; (A) plantar surface of the right foot, (B) anteroposterior X-ray view, (C) lateral surface of the right foot, and (D) profile X-ray view. Note the severe bone destruction caused by tumoral invasion.

**Figure 3:**
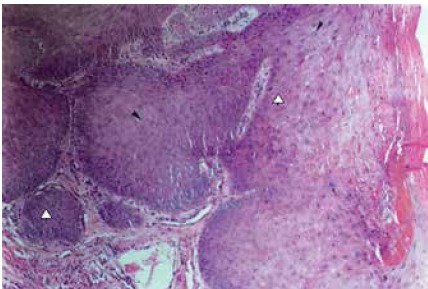
Case 1. Squamous cell carcinoma, ulcerative type. Photomicrograph of histological sample of the neoplasia, characterized by marked proliferation of cells in the epidermis, which appear as masses going deeper into the dermis (D). Note depolarization and mild cellular anomalies, characterized by occasional nuclear irregularity (λ) and prominent nucleoli (▸). Hematoxylin and eosin, 100x.

One (11.0%) patient presented osseous invasion, which was seen in simple X-rays of the affected foot. (Figure 2) In the other eight patients, the X-rays were normal. None of the two patients with metastasis to the regional lymph nodes presented tomographic alterations in the abdomen. Microscopically, the most frequent histological type was well-differentiated SCC, in eight cases (88.9%). (Figure 3) According to the TNM System for Malignant Tumor Classification from the Union for International Cancer Control (UICC), in six patients (66.6%) the primary tumor was type T1, in two patients (22.0%) was type T2, and one patient (11.0%) was type T3. The complete classification is shown in Table 3.

**Table 3:** Morphological and histological characteristics of tumors, and TNM classification for the study population.

Case	Morphological type	Histological type	Primary Tumor	Regional lymph nodes	Metastasis
1	U	PD	T2	N1	M0
2	V	WD	T1	N0	M0
3	V	WD	T1	N0	M0
4	U	WD	T1	N0	M0
5	V	WD	T2	N0	M0
6	U	WD	T1	N0	M0
7	V	WD	T1	N0	M0
8	V	WD	T1	N0	M0
9	U	WD	T3	N1	M0

Source: DOT-UFRJ, 2017. Abbreviations: U - ulcerative, V - verrucous, WD - well differentiated, PD - poorly differentiated.

Postoperative radiation therapy was not indicated for any patient. During the entire treatment period, the patients were monitored on an outpatient basis. The mean postoperative follow-up period was 51 months (range: 6-120 months). Two cases progressed to metastatic disease, leading to patient death (cases 1 and 9). Two cases recurred, and were treated with surgical revision and more proximal amputation (cases 3 and 6). There was no statistically significant relationship between initial definitive treatment and recurrence (p>5%). No patient developed postoperative complications requiring additional surgical intervention, such as infection or dehiscence of the surgical scar.

The mean MSTS score was 90% (varying from 80% to 100%), and all patients who were disease-free showed good or excellent functional results. There was no statistically significant relationship between initial definitive treatment and functional score (p>5%).

## DISCUSSION

SCC of the foot is a rare malignant tumor of the epidermis. Although this is the most frequent malignant tumor found in the soft parts of the foot, its incidence has been described as roughly 0.6 to 3.0%.^1^ ^,^ ^7^ ^,^ ^12^ ^,^ ^13^ It is commonly associated with chronic sun exposure, due to elastic degeneration of the dermis, irregular pigmentation, and telangiectasia.^14^ ^,^ ^18^ ^,^ ^19^ However, SCC can also develop on ulcers, chronic granulomas, and fistular sinuses after ingestion of arsenic and exposure to tobacco smoke or radiation.^9^ ^,^ ^12^ ^,^ ^14^ ^,^ ^20^ In broad terms, affected patients are in their 50s and 60s, and the disease affects men more frequently than women, at a ratio of 3:1.^2^ ^,^ ^12^ Caucasians are more likely to develop SCC.^7^ ^,^ ^10^ In this study, eight of the nine patients were men, the mean age at diagnosis was 60 years, and all participants reported daily exposure to ultraviolet radiation, which is common in tropical countries; three patients also smoked more than one pack of cigarettes per day.

The diagnosis is based on a high index of suspicion. Any changes in color, shape, or size, or sudden onset of pain in benign lesions of the foot should raise concerns about possible malignancy.^11^ Furthermore, particular attention is recommended for any chronic lesion that has difficulty healing. Incisional biopsy should be performed first for definitive confirmation of the diagnosis;^10^ scrape biopsy is not considered appropriate.^2^ Microscopically, there is disruption of the basal membrane and dermis invasion by well-differentiated keratinocyte-like cells. Corneal pearls may be present. An increase in the number of atypical cells, greater degree of anaplasia, and increased number of mitotic figures indicate little differentiation of the tumor.^2^ ^,^ ^12^ In some cases, the basal membrane remains intact and the carcinoma develops *in situ*.^2^ ^,^ ^6^

Treatment is based on surgical resection of the tumor. In general, lesions with regular margins should be treated with local excision.^2^ ^,^ ^6^ ^,^ ^8^ ^-^ ^10^ ^,^ ^18^ Safety margins of at least 3.0-5.0 mm should be maintained, in accordance with the dermatological literature.^6^ In more aggressive lesions, it is difficult to establish safety margins, and consequently more proximal amputation of the foot is indicated.^9^ ^,^ ^10^ Complete excision of the tumor rarely leads to recurrence. In our study, two patients experienced recurrence, probably due to inadequate resection of the primary lesion, and required a higher level of amputation (cases 3 and 6). In untreated cases, SCC of the foot spreads to the regional lymph nodes.

The prognosis is determined by the presence of metastasis, lymph node involvement, treatment, and local recurrences. There is generally greater potential for metastatic disease when SCC develops on chronic ulcers, known as Marjolin ulcers. SCC is spread hematogenically as well as through the lymphatic vessels, depending on its location and its degree of aggressiveness.^7^ ^,^ ^12^ In general, metastasis to the lymph vessels without bone or tendon involvement is extremely rare. But when ganglionic metastasis does occur, approximately 31% of patients die within five years.^9^ ^,^ ^19^ ^,^ ^20^ In the present study, two patients developed metastatic disease, and died from related causes.

Prophylactic radiation of the inguinal lymph nodes and intraarterial infusion of the affected limb with methotrexate and floxuridine have been suggested after excision of the primary tumor.^9^ However, prophylactic lymph node resection is controversial. Glass et al. recommend palpating the inguinal lymph node chain for 36 months after tumor excision.^20^ Lymph node biopsy should always be performed after this period if enlarged or painful lymph nodes persist.^9^

## CONCLUSIONS

Epidermoid carcinoma in the foot region is generally diagnosed late and subject to non-optimal initial treatment, requiring radical procedures with compromised functional results. Risk factors that can be identified early are present in the majority of patients. Biopsy in suspected cases and the use of oncologic principles can avoid errors in diagnosis and treatment. In our series, despite late diagnosis and surgical treatment with partial and total amputation of the foot, good oncological results were seen, which avoided systemic spread of the disease and provided the desired functional outcomes.
